# Cholinergic Modulation of Locomotor Circuits in Vertebrates

**DOI:** 10.3390/ijms231810738

**Published:** 2022-09-14

**Authors:** Didier Le Ray, Sandrine S. Bertrand, Réjean Dubuc

**Affiliations:** 1 Institut des Neurosciences Cognitives et Intégratives d’Aquitaine (INCIA), UMR 5287, Université de Bordeaux-CNRS, F-33076 Bordeaux, France; 2 Department of Neurosciences, Université de Montréal, Montréal, QC H3C 3J7, Canada; 3 Department of Physical Activity Sciences and Research Group in Adapted Physical Activity, Université du Québec à Montréal, Montréal, QC H3C 3P8, Canada

**Keywords:** acetylcholine, neuromodulation, locomotion, descending control, brainstem, spinal cord, mesencephalic locomotion region (MLR)

## Abstract

Locomotion is a basic motor act essential for survival. Amongst other things, it allows animals to move in their environment to seek food, escape predators, or seek mates for reproduction. The neural mechanisms involved in the control of locomotion have been examined in many vertebrate species and a clearer picture is progressively emerging. The basic muscle synergies responsible for propulsion are generated by neural networks located in the spinal cord. In turn, descending supraspinal inputs are responsible for starting, maintaining, and stopping locomotion as well as for steering and controlling speed. Several neurotransmitter systems play a crucial role in modulating the neural activity during locomotion. For instance, cholinergic inputs act both at the spinal and supraspinal levels and the underlying mechanisms are the focus of the present review. Much information gained on supraspinal cholinergic modulation of locomotion was obtained from the lamprey model. Nicotinic cholinergic inputs increase the level of excitation of brainstem descending command neurons, the reticulospinal neurons (RSNs), whereas muscarinic inputs activate a select group of hindbrain neurons that project to the RSNs to boost their level of excitation. Muscarinic inputs also reduce the transmission of sensory inputs in the brainstem, a phenomenon that could help in sustaining goal directed locomotion. In the spinal cord, intrinsic cholinergic inputs strongly modulate the activity of interneurons and motoneurons to control the locomotor output. Altogether, the present review underlines the importance of the cholinergic inputs in the modulation of locomotor activity in vertebrates.

## 1. Introduction

The organization of the neuronal structures responsible for generating and controlling locomotor activity among vertebrate species is conserved remarkably well. Comparable neuronal mechanisms operate from agnathans to humans [1,2]. The basic muscle synergies responsible for body propulsion are generated by spinal cord neurons, collectively known as “central pattern generators (CPGs)” for locomotion (for review see [1,3,4,5,6,7,8]). In turn, the spinal CPGs are activated and controlled by supraspinal structures that play a crucial role in starting, maintaining, and stopping locomotion [9,10] as well as controlling speed and direction (reviewed in [11]).

Sensory inputs play a crucial role in adapting locomotor activity to prevailing external and internal conditions [12,13]. Significant advances have been made in characterizing how sensory inputs act on spinal cord neurons and supraspinal structures to control locomotion and in defining the neural mechanisms involved (reviewed in [1,5,13,14,15]).

The supraspinal structures controlling locomotion are in large part organized in a serial fashion. Reticulospinal neurons (RSNs) give rise to descending pathways, comprising axons that make direct and indirect connections with interneurons and motoneurons in the spinal cord [16,17]. Several populations of RSNs have been identified and found to play a crucial role in starting, maintaining, and stopping locomotion [9,10,18,19] (for review see [20]).

The RSNs receive inputs from brainstem and forebrain regions, some of which are specifically dedicated to the control of locomotion. Locomotor regions include the mesencephalic (MLR) and diencephalic (DLR) locomotor regions [21,22,23,24,25]. The DLR corresponds to the zona incerta in the diencephalon [26], and its detailed connections with other forebrain and brainstem regions are not fully understood. In lampreys, DLR neurons have been shown to make monosynaptic glutamatergic connections with downstream RSNs [27]. The MLR was first discovered in the cat in the mid 1960s by the group of G. Orlovsky [28]. Since then, the MLR has been found to be present in all of the vertebrate species examined to date. It controls locomotion through connections to the RSNs [23,29,30,31] (for a recent review, see [32]). The MLR is in turn under the influence of the basal ganglia [33] (for reviews see [1,34,35,36,37]).

As indicated above, sensory inputs play a crucial role in adapting locomotion to the external environment into which the animal is moving. To this end, RSNs receive both direct and relayed sensory information from multiple sensory modalities, and this allows them to generate an adapted descending motor command to the spinal locomotor networks [38,39]. As of now, sensory inputs to RSNs have been shown in the lamprey to originate from skin mechanoreceptors, and from vestibular, visual, and olfactory receptors [15,40,41,42,43,44].

Several neurotransmitter systems modulate the neural circuitry controlling locomotion. Cholinergic neurons intervene along the locomotor control chain by acting on supraspinal and spinal neurons. In vertebrates, acetylcholine (ACh) is also involved in the primary developmental processes such as cell proliferation, migration, growth, and differentiation (for a review see [45]). Cholinergic neurons and ACh receptors appear in the developing neuronal networks during embryonic life. In the rat for example, the two families of ACh receptors, namely the nicotinic and muscarinic ACh receptors (respectively nAChRs and mAChRs), are detected early in the brainstem and in the spinal cord. The nAChRs appear at the embryonic day 12 (E12), whereas the mAChRs at E16. Thereafter, both nAChRs and mAChRs progressively emerge in the more anterior brain regions of the mesencephalon/diencephalon and neocortex during the E14-18 and E18-22 prenatal periods, respectively [46,47].

Amongst a large variety of roles that ACh may have during the animal’s life, it is involved in general motor functions, from the generation of supraspinal motor commands to the activation of muscles in the periphery. ACh plays also an important role in cortical arousal and, consequently, it modulates attentional [48,49,50] and motivational processes (nAChRs: [51]; mAChRs: [52]). The latter effects were proposed to impact motor planning in humans [53]. There are several examples of ACh modulation in the CNS that are not limited to motor function. In this review, we will focus on the mechanisms directly linked to the control of movement, and more specifically, locomotion.

## 2. Brainstem Cholinergic Mechanisms Controlling Motor Activity

### 2.1. The MLR Contains ACh Neurons

Large populations of cholinergic neurons are present in the caudal mesencephalon and rostral pons, in regions traditionally associated with the location of the MLR. The initial studies describing the MLR in decerebrate cats [28], revealed that the cuneiform nucleus (CuN) was part of this locomotor region (for reviews see [21,54]). The most striking observation at the time was the tight coupling between the MLR stimulation strength and the intensity of the locomotor output that ensued: as the MLR stimulation intensity increased, the generated locomotor speed also increased proportionally, as if locomotion was controlled by a rheostat. The initial discovery of the MLR ignited significant new interest in the supraspinal control of locomotion. Several animal species became research subjects in the field, and the MLR was functionally identified in all of the vertebrate species in which it was investigated (reviewed in [35,54]). Perhaps the most noticeable observation was the similar location of the MLR in those vertebrate species. Moreover, it was found that the MLR projections were very similar (reviewed in [55]). In the cat, the comparison between the distribution of the choline acetyltransferase-labeled cells (according to Kimura et al. [56]) and the regions that efficiently induced locomotion upon their electrical stimulation [57,58] showed a striking similarity [59]. It is now generally accepted that the MLR contains a mixture of glutamatergic, GABAergic, and cholinergic neurons distributed over several adjacent brainstem nuclei.

The pedunculopontine nucleus (PPN) was also shown in rats to be part of the MLR thanks to the elegant work of E. Garcia-Rill [60,61] (for reviews see [62,63]). The CuN and PPN occupy a large part of the mesencephalon, and, therefore, it is likely that the different parts of these two nuclei could be associated with different aspects of locomotor control. Additionally, it was shown in both lampreys and salamanders, that the laterodorsal tegmental nucleus (LDT) also controlled locomotion efficiently. The LDT is located medially near the caudal pole of the PPN, and it contains a large proportion of cholinergic neurons. In lampreys and in salamanders, the LDT was shown to be the most efficient region from which locomotion could be elicited and controlled [24,64] (for reviews see [37,54]).

It appears therefore that the PPN and CuN, and the LDT, at least in basal vertebrates, constitute the main brainstem nuclei responsible for the locomotor effects of the MLR stimulation (e.g., [24,60,64,65]). In addition, recent work carried out on subjects with Parkinson’s disease [66] showed that the mesencephalic deep brain stimulation of the PPN and CuN areas efficiently treated the freezing of gait, suggesting that the PPN and CuN may also be constitutive of the MLR in humans. These nuclei project to a variety of brain areas [67], and contain different proportions of glutamatergic and cholinergic projection neurons [68,69,70,71].

### 2.2. MLR Implications in Locomotor Control: Targets, Pathways, and Pharmacology

Imaging experiments in humans revealed that both the CuN and the dorsal PPN are active during imaginary fast walking [72]. Cholinergic neurons of the PPN have also been proposed to be involved in several motor functions including REM sleep, cervical tone, startle responses and locomotion (reviewed in [73]). In primates including humans, PPN cholinergic neurons play a role in controlling gait and posture [72]. Similarly, activating the ventral PPN, which mostly contains cholinergic neurons in cats, suppresses tonic muscle tone, subsequently allowing locomotion [50], whereas the genetic suppression of the vesicular ACh transporter in cholinergic neurons of both the PPN and LDT generates dramatic motor deficits in mice [74]. In contrast, it was shown that a complete excitotoxic lesion of the PPN alone failed to affect gait significantly in rats [75].

The MLR sends cholinergic axons to many key forebrain and brainstem structures controlling locomotion [67]. Therefore, it is likely that the MLR constitutes the major source of ACh modulation for locomotion. The basal ganglia regulate goal-directed behaviors by exerting an inhibitory control on the MLR, as shown by experiments in which a MLR disinhibition allowed for the initiation of locomotion (see [1]). In turn, MLR neurons project to the basal forebrain structures involved in locomotor regulation such as the basal ganglia [76,77] and the ventral tegmental area (VTA; e.g., [78]). Indeed, in mammals, the PPN and LDT cholinergic neurons projecting to the VTA were proposed to modulate locomotion related to drug- and novelty-seeking behaviors, specifically (reviewed in [79]) via both mAChRs [52] and nAChRs [51]. In rats, the optogenetic activation of PPN cholinergic neuron terminals in the VTA increases locomotion, whereas the activation of cholinergic terminals from the LDT tends to reduce locomotion [78] (but see also [80]). These effects presumably result from modulatory effects exerted by the VTA on the striatum.

Stimulation of the PPN in the rat triggers a dopamine release in the basal ganglia through the activation of both ionotropic glutamate receptors and nAChRs, and through the activation of mAChRs, all found on substantia nigra neurons. On the other hand, the M2-type muscarinic autoreceptors located on PPN neurons decrease the nigrostriatal dopamine [76]. In addition, ACh interneurons are present in the striatum where they exert a counterbalancing effect to dopamine inputs, thus regulating the striatum activity (reviewed in [81]). Modeling experiments suggested that this interaction is necessary to induce locomotion [82]. In contrast, the cholinergic innervation originating in the PPN was proposed to allow the basal ganglia to modulate/adapt ongoing locomotion to behavioral constraints (reviewed in [83]). Nevertheless, whatever the source of ACh (local interneurons within basal ganglia or PPN neurons), interactions between ACh and dopamine in the striatum appear essential for motor control, and motor dysfunctions in Parkinson’s disease have been correlated with the dysregulation of such interactions (e.g., [72,84]; for a review, see [81]). Although an increasing amount of data illustrate the impact of the MLR stimulation on forebrain motor structures, the control exerted by the MLR on locomotion is largely associated with the regulation of downstream motor nuclei of the brainstem (reviewed in [35,54]).

The cholinergic neurons seem to represent only about one quarter of the PPN neurons projecting to the reticular nuclei in mice [19]. In contrast, various studies in another rodent species, the rat, showed that most descending PPN neurons [85,86] and a substantial part of the reticular cell-connecting CuN neurons [87] are cholinergic. It is noteworthy that rat CuN cholinergic neurons are involved most likely in sensory modulation and cardiovascular regulation [87], which suggests a secondary role for these cholinergic neurons in locomotor control in this species. Therefore, recent optogenetic studies in rodents have attempted to establish the specific contribution of the glutamatergic and cholinergic neuronal populations in the MLR to the control of locomotion.

Glutamatergic cells in the CuN were shown to play a role in generating the locomotion at different speeds [88,89,90,91], whereas glutamatergic neurons of the PPN rather control locomotion at lower speeds [89,90]. In some cases, locomotion was halted by activating PPN glutamatergic neurons [90,92,93], and the induction or termination of locomotion was shown to depend on the respective activation of the glutamatergic and GABAergic reticulospinal systems of the lower brainstem [19]. Such a MLR-controlled start/stop system seems remarkably well conserved in vertebrates since comparable results were found in the lamprey [10]. The same approach has been used to examine the role of the PPN cholinergic neurons in the control of locomotion. As of now, the results are contradictory as increases and decreases in locomotor output were observed [88,89,90].

All these results strongly suggest that glutamatergic MLR neurons play a predominant role in triggering locomotion through the activation of downstream RSNs compared to cholinergic MLR neurons [94]. This is supported by the observation in cats that cholinergic antagonists seem to produce only little and temporary effects on spontaneous [95] or MLR stimulation-induced locomotion [96]. In striking contrast however, the direct injection of ACh agonists in the reticular formation is sufficient to trigger locomotor bouts in rats, birds, and lampreys [64,97,98], while ACh antagonists block MLR-induced RSN depolarization and locomotion in rats [97,99]. The latter results indicate that ACh may also play a significant role in MLR-Induced locomotion. Therefore, activating either the glutamatergic or cholinergic system independently may yield an incomplete picture of the specific role of these two neurotransmitter systems in controlling locomotor behavior. In addition, because slight variations in the experimental approaches (stimulation site, intensity/frequency parameters) may trigger different motor performances (e.g., [100]) a degree of caution is required in the interpretation of the behavioral observations resulting from localized brain stimulation.

Recently, the group of Bretzner [90] has shown in mice that the joint activation of glutamatergic and cholinergic neurons in the PPN modulated CuN-evoked locomotion, converting running into walking, thereby suggesting that both the glutamatergic and cholinergic systems participate in the MLR command. Interestingly, neurotransmitter interactions in the context of the MLR-controlled locomotion has already been reported in the lamprey, and it may be conserved across vertebrates [36], where forebrain-originating dopamine enhances both the MLR command onto RSNs [33] and RSNs directly [101]. Both the glutamatergic and cholinergic MLR inputs were shown electrophysiologically in the lamprey to converge onto RSNs, and each transmitter system can trigger a sustained RSN depolarization associated with locomotion in a semi-intact preparation (reviewed in [35]). Therefore, analyzing the RSN responses to both the glutamatergic and cholinergic MLR inputs is necessary to eventually understand the exact contribution of each neurotransmitter in the MLR-evoked locomotion in mammals.

### 2.3. Cholinergic Effects on Reticulospinal Neurons

**Figure 1:**
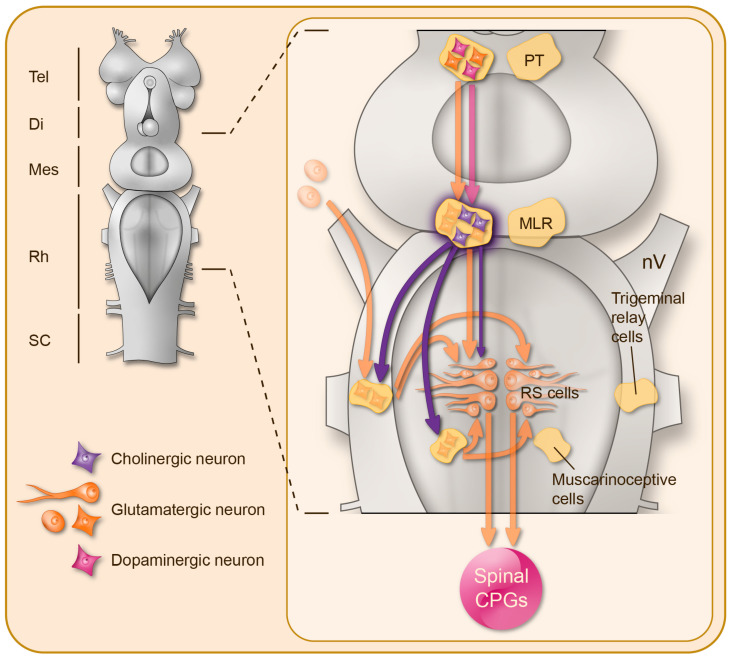
Cholinergic control of the reticulospinal neurons in the lamprey brainstem. **Right**: Schematic representation of the lamprey CNS, illustrating from top to bottom: the forebrain (Tel: telencephalon; Di: diencephalon), the brainstem (Mes: mesencephalon; Rh: rhombencephalon), and the spinal cord (SC). **Left**: Magnification of the brainstem illustrating the distribution of the cholinergic neurons (purple) and their projections. Under the control of glutamatergic (orange) and dopaminergic (pink) descending inputs from the posterior tuberculum, the mesencephalic locomotor region exerts glutamatergic and nicotinic cholinergic excitation on the reticulospinal (RS) neurons that in turn activate the downstream spinal central pattern generators for locomotion (CPGs). There is also a muscarinic cholinergic neuromodulation onto a population of excitatory ponto-medullary muscarinoceptive neurons, which in turn boost the reticulospinal neuron activation, and onto the trigeminal relay cells in order to filter the sensory inputs conveyed by the trigeminal nerves (nV) to the reticulospinal neurons.

Whereas the cholinergic effects of the MLR on other supraspinal structures is still not understood at the cellular level, the lamprey model has allowed researchers to gain a better understanding about the MLR-related ACh effects on downstream RSNs in the context of goal-directed locomotion (Figure 1).

The local application of ACh or nicotine on pontine reticulospinal nuclei (MRRN) consistently produced excitatory postsynaptic potentials (EPSPs) in a dose-dependent fashion in the RSNs. Large doses triggered locomotor activity in either isolated brainstem/spinal cord (fictive locomotion in spinal ventral roots) or in semi-intact preparations (active locomotion) [64]. In addition, the ACh-evoked EPSPs showed summation properties coherent with the known graded effect of the MLR stimulation. Moreover, during spontaneous swimming in a semi-intact preparation, both nicotinic agonists significantly accelerated the ongoing rhythm [64]. As the first direct demonstration of ACh inputs on RSNs, this indicates that the ACh inputs from the MLR are likely to play a substantial role in the initiation and the control of locomotion in lampreys, by allowing the pontine RSNs to generate sustained depolarization and firing under cholinergic effects. In lampreys, the pontine RSNs and their activation by ACh are crucial for MLR-induced locomotion, while the bulbar RSNs (PRRN) seem to produce less powerful effects, since only the glutamatergic antagonists prevented the acceleration of the locomotor rhythm by a stronger MLR stimulation [102]. The latter finding underlines the major contribution of the glutamatergic MLR inputs onto the bulbar neurons of the lateral paragigantocellular nucleus reported in mammals [94]. These similarities between lampreys and mammals certainly warrant a much needed, redirected attention to the pontine RSNs in mammals.

### 2.4. A Parallel Muscarinic Hyperdrive to Boost the Locomotor Output

As indicated above, the MLR projections to RSNs are essential to control locomotion. In lampreys, the MLR projects directly to RSNs via monosynaptic glutamatergic and cholinergic connections, and it also projects to RSNs indirectly via cholinergic connections (Figure 1). The direct cholinergic excitation is nicotinic in nature [64], while the indirect projection is muscarinic [103]. A bath application of muscarine elicits sustained and recurring depolarizations in RSNs. Calcium imaging revealed oscillations in calcium levels that occurred synchronously within the entire RSN population that was imaged. These oscillations were abolished by the bath application of TTX indicating that they were not intrinsic but driven by other neurons. Subsequent experiments revealed that such RSN oscillations were driven by a group of cells located at the junction between the pontine and bulbar reticular formation. The driving cells were named “muscarinoceptive”, and anatomical studies revealed that these neurons project directly to the reticular formation. The resulting effect of activating this group of cells is a marked amplification of the activity in the RSNs and an increase in the duration of locomotor output. The muscarinoceptive cells were shown themselves to respond to muscarine with long-lasting bouts of activity, to receive a direct muscarinic excitation from the MLR, and to send a glutamatergic excitation to the RSNs. Blocking the mAChRs on these neurons dramatically reduced the MLR-induced excitation of RSNs and slowed locomotion. These results further explain the previous observations in the lamprey that muscarine elicited a sustained depolarization in RSNs [104].

The presence of such a boosting mechanism has yet to be demonstrated in other vertebrate species. However, findings from older studies revealed that the activation of muscarinic receptors in the same region of the reticular formation in birds and mammals can elicit bouts of locomotion. Indeed, the locomotor behavior in birds can result from brainstem injections of carbachol, a nonspecific cholinergic agonist, and these effects are blocked by the muscarine receptor antagonist, atropine [98]. Cholinergic inputs are also believed to activate brainstem neurons in mammals [97,99], and a group of muscarine-sensitive neurons receiving cholinergic inputs from the PPN was described in rats in the ventromedial medulla close to the pontine border [105], at a location similar to that of the muscarinoceptive cells in lampreys. The role of these neurons was not described in relation to locomotion in mammals, but the similarities in their properties and location support the idea that they could amplify the reticulospinal descending signals to boost the locomotor output, just as has been observed in lampreys.

The presence of muscarinic effects in birds and mammals, as in lampreys, suggests that the muscarinic amplifying mechanism is conserved in evolution. It further suggests that the supraspinal control of locomotion in vertebrates is not exclusively composed of linear projections down from the forebrain to the spinal cord. Rather, there exists an additional feedforward cholinergic “hyperdrive” component, originating in the MLR and allowing for a supplementary activation of the locomotor system.

### 2.5. Muscarinic Control of Brainstem Sensory Inputs

In lampreys [40,41] as in mammals (e.g., [106]) the reticular neurons, and especially the RSNs, integrate information from various sensory modalities in order to generate adaptive motor commands. It is also well established that cat RSNs that are rhythmically active during locomotion, in addition receive sensory inputs from a large portion of the body [107]. Interestingly, the MLR has been shown to modulate sensory inputs that reach the RSNs in the lamprey [108] (Figure 1). Indeed, a short duration electrical stimulation of the MLR depresses, for several tens of minutes, the EPSPs evoked in RSNs by trigeminal nerve stimulation. These effects were prevented by perfusing the brainstem with the muscarinic antagonist atropine. Moreover, mAChRs were immunohistochemically identified on the cells that relay trigeminal sensory signals to the RSNs [109], as well as on the RSNs themselves. Similar muscarinic effects were observed after ejecting ACh or the muscarinic agonist pilocarpine directly onto intracellularly recorded RSNs while electrically stimulating the trigeminal nerve [110]. This muscarinic modulation was not related to any changes in the RSN membrane properties, but rather depended on a presynaptic control of the synaptic transmission between the trigeminal relay cells and RSNs. In contrast, the perfusion of atropine strongly potentiated the RSN responses to trigeminal nerve stimulation, suggesting the presence of a tonic inhibitory mAChR-mediated regulation of RSNs and/or trigeminal relay cells. These observations in lampreys suggest that predictable sensory inputs would be gated by MLR inputs, allowing for a smooth behavioral output to occur during goal-directed locomotion. Such a filtering has been proposed in numerous species for phasic sensory inputs impinging on the locomotor CPGs in the spinal cord where there is a strong presynaptic modulation of the primary afferent terminal inputs during fictive locomotion (see below and [111]), but was not envisioned yet in the brainstem. In addition, when the MLR is inactive the muscarinic modulation would be strongly decreased, and this would open the gates for sensory inputs and thus facilitate sensory-evoked locomotion (e.g., escape swimming [40,41]). Moreover, blocking the muscarinic inputs unmasked persistent RSN membrane potential oscillations after applying NMDA onto the RSNs [110] that could further support escape swimming.

## 3. Cholinergic Mechanisms in the Spinal Cord

**Figure 2:**
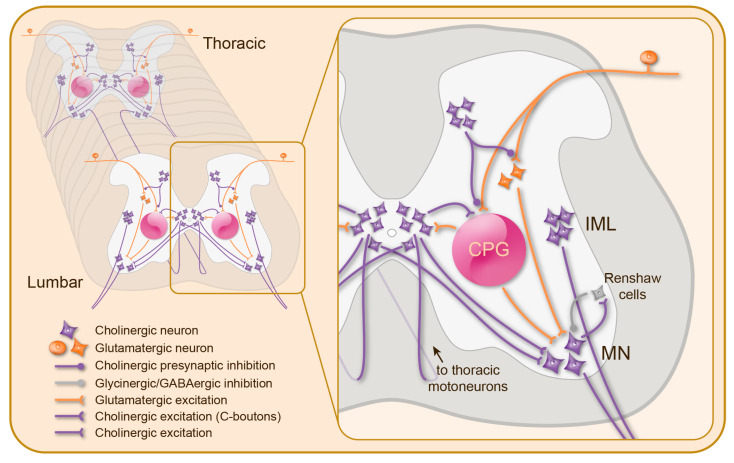
Cholinergic neuromodulation of spinal motor networks. Motoneurons (MN), intermedio-lateral neurons (IML), and propriospinal interneurons are the only sources of acetylcholine (purple) in the spinal cord, while local glutamatergic excitation (orange) is provided by segmental sensory afferents and interneurons, some of which are part of the central pattern generator (CPG) for locomotion. Except for a group of dorsal interneurons that perform a presynaptic inhibition (filled circle) onto the primary afferent terminals, all propriospinal cholinergic interneurons exert excitation on a variety of cellular targets, including motoneurons (notably via the well described C-boutons) and CPG interneurons.

Cholinergic neuromodulation plays a crucial role in spinal cord function. The spinal cholinergic system (Figure 2) is exclusively intrinsic to the spinal cord (propriospinal) and the cholinergic modulation results from the activity of a relatively small number of spinal neuronal populations [112]. Among them, the somatic motoneurons (MNs) play a key role during spinal cord early development by releasing ACh onto their neighboring cells, which triggers spontaneous motor bursts that spread over the entire spinal cord (reviewed in [113]). This bursting activity allows the motor networks and neuronal phenotypes to develop properly [114,115,116,117]. Indeed, in the spinal cord, most of the cholinergic cells are MNs that innervate skeletal muscles, with their cell bodies distributed within the ventral gray matter throughout the spinal cord. In mammals, the preganglionic cells (e.g., [118]), a population of “motoneuron-like” neurons with axons in the ventral roots are located in the thoracic and lumbar segments and also use ACh as their neurotransmitter. In addition, various populations of cholinergic interneurons (INs) have also been described, such as small neurons distributed within the dorsal horn and around the central canal, as well as the large partition cells at the border between the dorsal and ventral gray matter [118,119,120]. Although the roles of these INs are less understood, it was suggested that they may constitute a major source of cholinergic neuromodulation in the spinal cord [121,122], notably involved in the control of spinally-generated locomotor activity [123]. Both nicotinic and muscarinic effects were described in the spinal cord.

### 3.1. Nicotinic Modulation of Spinal Motor Circuits

The nicotinic effects on locomotor networks were mainly attributed to the modulation of the activity of premotor INs [119,124,125,126] or direct effects on the excitability of MNs [119,126,127,128] in various vertebrate species, and lamprey experiments showed no evidence of a modulation of descending commands [129] or long distance-projecting INs in the spinal cord [130]. As observed in many species, MNs are depolarized in response to the nAChR activation [119,124,127,131], the origin of which remains unclear. MNs are known to also release ACh centrally, through axon collaterals terminating notably on the other synergistic MNs, as first demonstrated in adult cats [132,133] and later in other species (e.g., [125,128]). Some spinal cholinergic INs also project directly onto MNs [121,122] (Figure 2). Whereas in mammals, the cholinergic modulation of MN activity seems to be mostly linked with the activation of mAChRs (see below), the nicotinic effects on MNs are widespread in lower vertebrates such as the lamprey [119,126] and the amphibian *Xenopus laevis* embryo [124,134].

In addition to the local activation of MNs, the nAChR activation from MNs also plays a major role in the homeostatic modulation of the MNs’ own activity, through the activation of the Renshaw recurrent inhibition: when a MN is active, an axon collateral releases ACh, together with glutamate [135], to activate low affinity nAChRs on Renshaw cells [136,137]. The latter cells in turn inhibit MNs via glycinergic/GABAergic neurotransmission [138].

In rodents nAChRs are also present on primary afferent terminals in the dorsal horn [139,140], and a nicotinic modulation of somatosensory information has been found both in the periphery [141] and centrally on the primary afferent terminals [142,143]. The level of polarization of the terminals is now known to be continuously modulated and different mechanisms are involved. A depolarization of the afferent terminals has been classically associated with presynaptic inhibition [144,145,146] and ACh could play a role. In addition to membrane potential changes in primary afferent terminals, action potentials leaving the spinal cord via the dorsal roots (centrifugal sensory discharges) have been known to occur in vertebrates [147]. Such antidromically conducted discharges occur spontaneously or may result from the activation of other sensory afferents, or from the central neural networks [111,148]. Antidromic discharges can also be elicited by the MN activation itself [149]. However, although a direct modulation of sensory afferents by the MNs was extensively described in an invertebrate [150,151] it is unlikely in vertebrates that such discharges result from direct connections from MN axons onto the primary afferent terminals, but more likely from the involvement of spinal inhibitory interneurons co-releasing GABA and ACh [112,143]. Because the primary afferents are responsible for the fast adaptation of the locomotor activity to various behavioral constraints, a nicotinic modulation of local sensory inputs could obviously impact the function of the spinal motor circuits.

### 3.2. Muscarinic Modulation of Spinal Motor Circuits

In mammals, the cholinergic neuromodulation of the spinal networks is mainly achieved through muscarinic effects with a contribution of several of the mAChRs described so far (for a recent review, see [152]). Originating from the V0c INs, most likely part of the medially-located partition cells, the well characterized C-bouton synapses constitute one source of muscarinic modulation onto MNs (structure and function reviewed in [152,153]; Figure 2). In the in vitro spinal cord preparation of neonatal rats, for example, enhancing the endogenous ACh activity by perfusing the cholinesterase inhibitor edrophonium, triggers episodes of organized fictive locomotion, the pattern of which is comparable to that of active locomotion [96]. An exhaustive analysis of the cholinergic pharmacology demonstrated the lack of nAChR involvement and characterized the mAChRs responsible for such an endogenous neuromodulation and their actions on both MNs and local INs [96]. Interestingly, previous studies in rat neonates also showed some kind of motor activation in lumbar spinal segments in response to the application of exogenous ACh [154], although the extensor/flexor alternation characteristic of locomotion was not observed. These effects were insensitive to large spectrum nAChR antagonists, but were prevented by the classical mAChR blocker, atropine.

Muscarinic agonists exert various, and sometimes opposite effects on the spinal networks. For instance in mice, the endogenous activation of type-3 receptors enhances the amplitude of glutamatergic miniature potentials recorded from MNs and stabilizes the locomotor-related bursting, whereas the type-2 receptors mediate the ACh-induced decrease of glutamatergic miniature potentials while increasing the fictive locomotor frequency and burst amplitude [155]. The link between the changes in miniature potential amplitude and the effects on locomotion remains to be determined. The source of the glutamatergic miniature potentials was not investigated in detail but they could originate, at least partly, in the commissural interneuronal system, which has been demonstrated in rat neonates to be lastingly facilitated following the activation of motoneuronal mAChRs by local cholinergic INs in an atropine-sensitive manner [156]. Although type-2 and type-3 mAChRs exert distinct effects in the mouse spinal cord, these receptors do not seem to be present on the same pools of MNs [155], and a comparable MN subtype-specific muscarinic neuromodulation was also reported in zebrafish [157].

In many vertebrate species, MNs are the main targets of muscarinic modulation in the spinal cord, as mAChR activation globally results in an increase in MN excitability (see however [119,124]) via a large variety of cellular alterations. In urodeles, turtles and rodents, the mAChR activity tends to reduce several potassium currents in spinal MNs, such as the delayed rectifier and the M-current, as well as the hyperpolarization-induced cation current I_H_ and the calcium-activated potassium current responsible for the after-hyperpolarization I_AHP_ [155,156,158,159,160], all of which are responsible for bringing back or maintaining neurons in a hyperpolarized state. In contrast, voltage-gated calcium currents in turtle MNs are enhanced under muscarine, allowing for the generation and maintenance of longer plateau potentials [158,161,162].

Direct motoneuronal effects in combination with additional excitatory effects on spinal network INs [122,123] and local sensory afferents [140,141], result in a global excitation of motor networks under ACh neuromodulation. In the absence of a supplementary excitation provided by other neurotransmitters or neuromodulators, such as glutamate agonists or serotonin, the exogenous ACh (or muscarinic agonists) alone triggers slow non rhythmic ventral root motor bursts in newborn rodents [154] and adult turtles [161]. Those ventral root bursts were recently proposed in rodents to participate in the coupling between somatic and autonomic functions, as an intraspinal mechanism of coordination between the two neuronal functions [163]. In the presence of an additional excitatory drive, the cholinergic agonists rather modulate the ongoing locomotor rhythm in lampreys [126], *Xenopus* embryos [125], urodeles [164], and mammals (e.g., [155,156,160]).

## 4. Conclusions

Historically, ACh has been mostly associated with the synaptic transmission from motoneurons to muscles in vertebrates (see however [165]). It progressively became clear that a variety of neuronal populations widely distributed in the brain and spinal cord are cholinergic. The latter neurons are involved in numerous sensorimotor and cognitive functions conferring to the cholinergic system a broader role in the fundamental neuromodulation of neural activities, thus constituting a possible target of therapeutic approaches for a variety of neuronal and non-neuronal, motor and non-motor pathologies (e.g., [66,84,166,167,168]). For instance, the MLR, known to contain abundant populations of cholinergic neurons, is at present the target of new therapeutic approaches for Parkinson’s disease using deep brain stimulation. Currently, mixed effects have been found in patients [169,170], possibly due to the degeneration of MLR neurons in these patients as the disease progresses [171,172]. It has been proposed that deep brain stimulation of the MLR could also be used to ameliorate locomotor function after a partial spinal cord injury. At present, this approach appears to have excellent potential [173,174,175]. Further research is needed to improve our understanding of the cholinergic modulation in the brainstem and spinal cord.
